# Transformation with Artificial Chromosomes in *Oxytricha trifallax* and Their Applications

**DOI:** 10.1534/g3.119.400298

**Published:** 2019-10-14

**Authors:** Derek M. Clay, Hoyon Kim, Laura F. Landweber

**Affiliations:** * Departments of Biochemistry and Molecular Biophysics and Biological Sciences, Columbia University, New York, NY,; † Department of Molecular Biology, Princeton University, Princeton, NJ, and; ‡ Department of Biological Sciences, Columbia University, New York, NY

**Keywords:** Genome Rearrangement, Ciliates, Epigenetics

## Abstract

*Oxytricha trifallax*, like other ciliates, has separate germline and somatic nuclei. The diploid germline genome in the micronucleus is composed of long conventional chromosomes. The macronucleus contains a somatic genome which is naturally fragmented into thousands of kilobase-sized chromosomes. Here, we develop a method to stably incorporate artificial chromosomes into the macronucleus. We report two cases of successful transformation and demonstrate the use of somatic transformation to investigate gene regulation and gene function in *Oxytricha*. We show that the transformed artificial chromosomes are maintained through multiple asexual divisions. Furthermore, they support the transcriptional regulation of the native chromosome from which they were derived and are translated to produce functional proteins. To test if transformed chromosomes are amenable to practical applications, we generated a tagged version of a representative gene (AL1) and used it to co-precipitate associated proteins. This revealed an association with nucleic acid binding proteins, specifically RNA-binding proteins, and RNA immunoprecipitation of AL1 revealed its association with multiple RNAs. The use of artificial chromosomes in *Oxytricha* enables an array of genetic and molecular biological assays, as well as new avenues of inquiry into the epigenetic programming of macronuclear development and genome rearrangement.

The dual nuclear structure of ciliates provides an unusual challenge in the genetic manipulation of these model systems. In the hypotrich *Oxytricha trifallax*, the transcriptionally active macronucleus (MAC) is comprised of thousands of gene-sized nanochromosomes, on average 3kb long (Swart *et al.* 2013), maintained at high ploidy (reviewed in Prescott 1994; Yerlici and Landweber 2014). Though the ploidy of each gene-sized chromosome varies, transcription is only modestly correlated with gene copy number (Swart *et al.* 2013). This implies that the sequence of the nanochromosome, rather than copy number, may play a role in dictating the level of gene expression. However, *Oxytricha*’s nanochromosomes contain small noncoding regions (on average 107 bp upstream and 103 bp downstream of the gene) with potential regulatory sequences (Swart *et al.* 2013). Even with these short noncoding regions, evidence for regulation within them has been described for the Hsp70 nanochromosome in the related species *Oxytricha nova* by showing that upstream transcriptional heat shock elements are conserved (Anderson *et al.* 1996). This conventional sequence specific control of gene expression offers the opportunity for genetic manipulation, especially as only a single gene is typically present on a chromosome.

In essence, *Oxytricha*’s tiny chromosomes are fully functional units of a size amenable to *in vitro* synthesis (Beh *et al.* 2019). These kilobase sized chromosomes provide a potential avenue to artificially produce them and introduce them into the macronucleus. In *Euplotes* and *Stylonychia*, linear constructs have been introduced into the vegetative macronucleus through microinjection and lipid transfection. These constructs were stably maintained at high copy levels and transcriptionally active (Bender *et al.* 1999, Erbeznik *et al.* 1999, & Skovorodkin *et al.* 1999). In *Euplotes crassus*, nanochromosomes containing the TATA-binding protein gene replaced with a neomycin resistance gene, and the rDNA chromosome with an anisomycin resistance mutation have been successfully transformed and confer antibiotic resistance to the transformed cells (Bender *et al.* 1999, Erbeznik *et al.* 1999). In *Stylonychia lemnae*, the α-Tubulin minichromosome has been successfully transformed into vegetative cells and the authors demonstrated the importance of telomeric sequences for the transformant to be stably maintained (Skovorodkin *et al.* 1999, Skovorodkin *et al.* 2001). In addition to proof of concept, further studies transformed multiple variations of the α-Tubulin minichromosome to identify promoter elements upstream of the α-Tubulin gene (Skovorodkin *et al.* 2007).

One protein domain of interest in *Oxytricha* is the Alba domain and related proteins. In *Oxytricha*, there is evidence that some of these Alba-like proteins may have a role in post-zygotic development of *Oxytricha trifallax*. The Alba-like 1 (AL1) and Alba-like 2 (AL2) genes are among the most highly expressed genes during early conjugation in *Oxytricha trifallax* (Swart *et al*. 2013). In the closely related ciliate, *Stylonychia lemnae*, the macronuclear development protein 2, an Alba domain protein, is similarly highly expressed in conjugation (Fetzer *et al.* 2002). When dsRNA against MDP2 was fed to *Stylonychia lemnae*, the cells stalled during sexual development (Bulic *et al.* 2013). Further characterization of Alba domain proteins has been performed in other protists. In *Trypansoma brucei* and *Toxoplasma gondii*, Alba domain proteins have been shown to be involved in translation regulation (Gissot *et al.* 2013, Mani *et al.* 2011, Subota *et al.* 2011).

The genomic resources in *Oxytricha* are well established (Swart *et al.* 2013, Chen *et al.* 2014), as is the use of *Oxytricha* as a model system for studies of RNA biology and epigenetic inheritance, particularly during macronuclear development (Nowacki *et al.* 2008, Fang *et al.* 2012). However, to date there have been no studies reporting transformation in *Oxytricha*, likely due to the infrequent use of the model system. Here we describe the successful transformation of vegetative *Oxytricha trifallax* cells, along with the stable maintenance of constructs that are both transcriptionally and translationally active. In addition, we show successful applications of chromosome transformation to investigate biologically relevant questions in this model system.

## Methods

### Generation of DNA constructs for microinjection

To generate artificial chromosomes, PCR was performed on *Oxytricha* genomic DNA from the JRB310 strain or plasmid with a ciliate codon corrected enhanced GFP as template (Nowacki *et al.* 2005) using Phusion high-fidelity polymerase (NEB). These PCR products were then purified with MinElute columns using the PCR purification instructions (Qiagen). To stitch together the PCR products, purified PCR products with overlap (approximately 30-50 bp) at the ends were mixed at equal molar ratios with total DNA mass of 200 ng into a PCR reaction with Phusion high-fidelity polymerase (NEB) without any primers added. The PCR reaction was run for 15 cycles. Afterward, primers to amplify the stitched together product were added to the reaction and the PCR reaction was run for another 15 cycles. The correct constructs were purified by gel extraction with MinElute columns following manufacturer’s instructions (Qiagen). Purified constructs were A-tailed with Taq polymerase (Roche) and then TOPO-TA cloned (Invitrogen) according to manufacturer’s instructions. TOPO plasmids were transformed into TOP10 One shot chemically competent cells (Invitrogen) following manufacturer’s instructions. Plasmid DNA was harvested from clones using the QIAprep Spin Miniprep kit (Qiagen). Plasmids were Sanger sequenced by Genewiz.

The plasmids with verified insert sequences were used as template for PCR to produce 100 ug of synthetic chromosome with 20 base pair double stranded telomeres. The PCR products were then ethanol precipitated, resuspended in nuclease free water (Ambion) and put through ultra-free MC column (Millipore) according to manufacturer’s instructions to remove impurities. DNA constructs were quantified by QUBIT High Sensitivity DNA Assay kit (Thermofisher Scientific) for a final concentration of 1 to 3 mg/mL.

### Oxytricha culturing

*Oxytricha trifallax* cells were cultured in Pringsheim media (0.11 mM Na_2_HPO_4_, 0.08 mM MgSO_4_, 0.85 Ca(NO_3_)_2_, 0.35 mM KCl, pH 7.0) and fed with *Chlamydomonas reinhardtii* and *Klebsiella* according to previous published methods (Khurana *et al.* 2014). Cells of two compatible mating types (strains JRB310 and JRB510) were starved 12 hr to induce mating. Mating was initiated by mixing approximately equal numbers of starved cells from each type. Pringsheim was added to dilute the mating cells to a final concentration of 5,000 cells/mL.

Cells were encysted by filtering cells with cheesecloth, and then concentration by centrifugation (100g for 1 min). The cells were then resuspended in fresh Pringsheim media and left for three days in a petri dish without food. Cysts from the starved culture were concentrated by placing the culture into a graduated cylinder and allowing cysts to settle at the bottom of the cylinder. Liquid was aspirated off from the top and DMSO was added to a final concentration of 10% to the remaining cyst containing liquid. Cysts in 10% DMSO were stored at -80°. For excystment, cysts were thawed, washed three times with Pringsheim, and then fed with *Chlamydomonas reinhardtii* and *Klebsiella*.

### DNA transformation through injection

Vegetative *Oxytricha* cells were isolated and placed in Volvic brand mineral water with 0.2% BSA by mass. DNA constructs were injected into the macronuclei of the individual cells in the method described previously for paired cells (Nowacki *et al.* 2008). After injection, single cells were isolated into 1 mL of Volvic brand mineral water in individual wells on 24 well plates and were treated according to standard cell culturing methods mentioned above. As clonal population size grew, lines were transferred to 10 cm petri dishes and then grown in 1X Pringsheim media.

### Validation of transformation

*Oxytricha* lines were screened for successful transformation by using cells or purified genomic DNA as template in a conventional PCR. The ctg18685.0-GFP construct was verified by PCR using primers specific to the GFP sequence. For the rest of the constructs, an appropriate size shift in the PCR band (if the wild type nanochromosome was not present) or appearance of a second band of the expected size of was used to verify that the artificial chromosome was successfully transformed. Telomeric PCR on the Hsp70-GFP transformant were performed using a telomeric primer with linker sequence (AP12-C4A4; 15 cycles for each round with a 1:1000 dilution between rounds) and a GFP specific primer for the first round followed by a second round PCR using a linker primer (AP2) and nested GFP primers. Second round PCR products were gel extracted via MinElute gel extraction protocol (Qiagen) and sent for Sanger sequencing through Genewiz.

### Nucleic acid extraction and cDNA generation

Genomic DNA was harvested from *Oxytricha* through concentrating the cells by centrifugation for 1 min at 80g and then using the NucleoSpin Tissue kit (Macharey-Nagel) according to manufacturer’s instructions on the cell pellet.

RNA from cells was collected using the mirVANA miRNA Isolation Kit (Ambion) using the total RNA isolation protocol according to the manufacturer’s instructions. Isolated RNA was then DNAse-treated with Turbo DNAse (Ambion) following manufacturer’s instructions. cDNA was generated from RNA by using the SuperScript III First-Strand Synthesis kit (Thermofisher Scientific).

### Isolation of RNA From heat-shocked cells

Isolation of RNA for the heat shock experiment was done by concentrating 3 mL of cells via centrifugation (80g for 1 min), aspirating off media and resuspending in previous volume using temperature appropriate 1X Pringsheim (room temperature or 37°) and placed in a well of a 6 well plate. A preliminary heat shock time course was performed to determine the peak of the GFP transcript levels (data not shown). Based on the preliminary heat shock time course, cells were then incubated for 10 min at 37° or room temperature for 10 min, followed by RNA harvesting protocol above.

### Quantitative PCR on genomic DNA and cDNA

DNA and cDNA quantitation were performed using SYBR Green power mix (ABI) according to manufacturer’s instructions on a CFX384 (Bio-Rad) qPCR machine. Standards for qPCR were generated from conventional PCR purified by MinElute PCR purification (Qiagen). Standards were quantified by Qubit High Sensitivity DNA Assay Kit (Thermofisher Scientific) and diluted with nuclease free water (Ambion) to appropriate concentration for the standard curves. Results from qPCR assays were calibrated using the standard curves.

### Live cell imaging for GFP fluorescence after heat shock

We used a modification of the heat shock protocol listed above for RNA isolation (heat shock 20 min instead of 10 min). At the end of the heat shock, cells were spun down (80g for 1 min), media aspirated, and resuspended in 3mL of room temperature water and placed in a well of a new 6 well plate. The pools of cells were checked visually to confirm that the cells were fluorescent prior to isolating single cells. Exposure to light for excitation of the GFP did seem to increase movement of the cells; hence immobilizing the cells was required for imaging. Cells were left in the wells for 2 hr prior to isolation for imaging. Live *Oxytricha* cells were isolated on a glass coverslip and immobilized using the same protocol as for microinjections listed above. Cells were imaged by epifluorescence using an Axiovert 200 microscope (Zeiss) at 40x magnification with a mercury vapor lamp source (LEJ, EBQ 100) and the Zeiss filter set 10. Imaging was performed with and attached digital camera and Axiovision software (Zeiss).

### Transformed protein detection and isolation

To collect cleared cell lysate, matings (AL1-histidine tag transformant X JRB510 and JRB310 X JRB510) at 18 hr post mixing were concentrated by 10-micrometer Nylon Mesh (Small Parts) and further concentrated by centrifugation (100g for 1 min). Cleared cell lysate was generated from cell pellets after the aspiration of the supernatant. Cells were lysed with lysis buffer (lysis buffer: 10% glycerol, 1x protease inhibitor cocktail (Sigma), 1 mM PMSF, 1x Halt phosphatase inhibitor cocktail (Thermofisher Scientific), and 1 mM DTT in stock lysis solution) (stock lysis solution: 20 mM Tris pH 8.0, 150 mM NaCl, 2 mM EDTA, 1% NP-40) with 1 mL of buffer per 625,000 cells. Cell lysate was rotated at 4° for 30 min. Lysate was then spun down at 16,000g for 30 min. Supernatant was collected and flash frozen with liquid nitrogen prior to storage at -80°.

Western Blotting was performed on the cell lysates using the RM146 Anti-histidine tag antibody (Abcam) 1:2,000 with 1:3000 of Goat Anti-Rabbit IgG (H + L)-HRP Conjugate (#170-6515, Bio-Rad). Chemiluminescence was perform with Amersham ECL Western Blotting detection reagent (GE Healthcare) according to manufacturer’s instructions and imaged on silver film with a Kodak X-Omat film processor.

### Co-immunoprecipitation and mass spectrometry of histidine-tagged AL1 protein

Dynabead G (Invitrogen) was prepared with RM146 Anti-histidine tag antibody following manufacturer’s instructions. Cleared cell lysate derived from matings of 3 million cells was incubated with Dynabead G for 3 to 4 hr at 4° rotating before being washed 3X with IP wash buffer (lysis buffer without NP-40 detergent) and then 3X with volatile wash buffer (130 mM KCl, 50 mM NH_4_HCO_3_). Protein was eluted with NH_4_OH prior to being flash frozen with liquid nitrogen. For quality control, 20% of elution was collected and dried via speed vac before being resuspended in 1x SDS-PAGE buffer for gel analysis with Krypton staining (Thermo Fisher Scientific). A Typhoon scanner (GE) was used for imaging to ensure quality control. Mass spectrometry was done through the Princeton Mass Spectrometry Core facility.

### RNA-immunoprecipitation of histidine-tagged AL1 protein

18 hr post mixing cells for mating, approximately 3 million cells were concentrated with 10-micrometer Nylon Mesh (Small Parts) to a final volume of 36 mL. To the concentrated cells, 1 mL of formaldehyde (37%) was added to fix the sample, shaking the samples at room temperature for 10 min. To quench the fixation, glycine was added to a final concentration of 125 mM and shaken for 5 min at room temperature. The fixed cells were pelleted by centrifugation (500g for 1 min) and then washed twice with ice cold TBS. The pellet was then resuspended in RNA-IP lysis buffer (0.1% sodium deoxycholate, 1x Protease inhibitor, 1 mM PMSF, 1 mM DTT, and 0.1% RNAseOUT inhibitor in stock lysis solution), vortexed thoroughly, and then flash frozen with liquid nitrogen for storage at -80°.

Lysates were then sonicated (LE220 Covaris) for 5 min (450 W peak incident power, 10% duty cycle, 200 cycles per burst) for immunoprecipitation. Sonicated samples were centrifuged for 10 min at 16,000g to collect the supernatant with 10% (200 μL) being collected as input and flash frozen. To pull down the RNA, Dynabead G (Invitrogen) were prepared with RM146 Anti-histidine tag antibody following manufacturer’s instructions with the modification of 0.1% of RNAseOUT (Thermofisher Scientific) added to the PBST. Lysate was incubated with bead antibody conjugated beads overnight. Beads were washed twice at room temperature with each of the following buffers: buffer A (20 mM Tris pH 8.0, 2 mM EDTA, 150 mM NaCl, 1% Triton X-100, 0.1% SDS), buffer B (20 mM Tris pH 8.0, 2 mM EDTA, 500 mM NaCl, 1% Triton X-100, 0.1% SDS), buffer C (10 mM Tris pH 8.0, 250 mM LiCl, 1 mM EDTA, 1% NP-40, 1% sodium deoxycholate), buffer D (10 mM Tris pH 8.0, 1 mM EDTA). Proteinase K treatment was performed by adding 200 μl of 2x proteinase K buffer (200 mM Tris pH 7.5, 25 mM EDTA, 300 mM NaCl, 2% SDS) and 1 μl of 5 M NaCl to both the beads and the previously collected input samples. Crosslinking was reversed by incubating the samples at 65° for 2 hr. Samples were then treated with proteinase K (200 μg, Macharey-Nagel) for one hour at 60°. Afterward, RNA was acid phenol:chloroform (Ambion) extracted, ethanol precipitated, and resuspended in nuclease-free water (Ambion).

### Data Availability

Strains are available on request. Supplemental file 1 contains the nucleotide sequences from the Sanger sequencing. Supplemental file 2 contains the mass spectrometry results from co-immunoprecipitation of the histidine-tagged AL1 protein. Supplemental material available at FigShare: https://doi.org/10.25387/g3.9789434.

## Results

### Injected constructs are stably maintained during asexual growth

**Figure 1:**
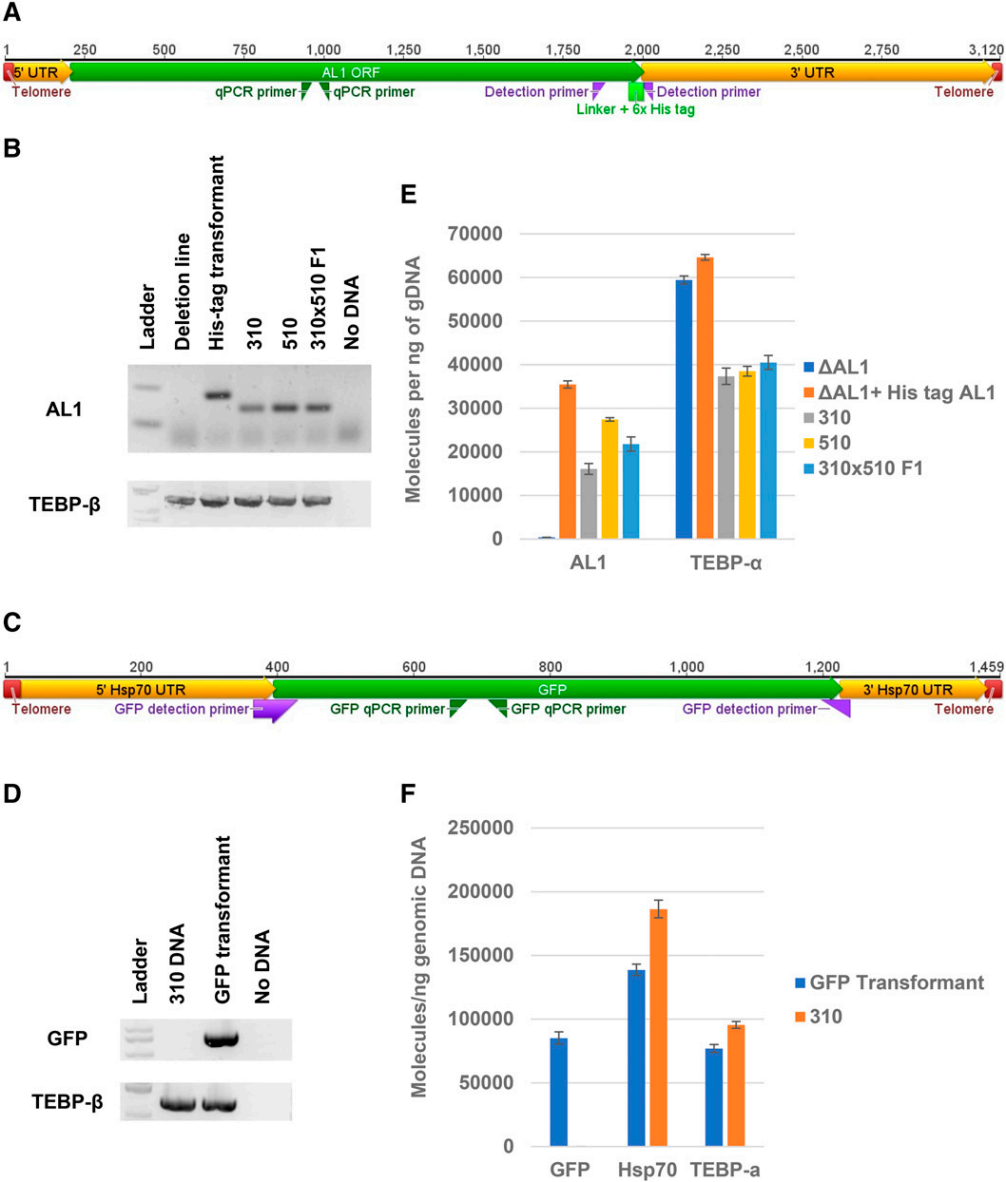
Detection and quantification of transformed nanochromosome in cell lines. Schematic maps of the transformed artificial chromosome AL1 with a C-terminal histidine-tag with the location of primers used to amplify the construct (A) and the artificial chromosome containing GFP in place of the Hsp70 gene (C). PCR amplification of genomic DNA collected from cell lines shows the successful transformation of the AL1 with a C-terminal histidine-tag (B) and the GFP constructs (D) using the detection primers. The AL1 construct was injected into the F1 progeny of JRB310 (labeled 310 in the figure) cells mated to JRB510 (510). These cells also contained a programed deletion of the entire AL1 chromosome. The GFP construct, on the other hand, was introduced into WT JRB310 cells. PCR of the native TEBP-β locus provided a loading control. Quantitative PCR shows the abundance of the transformed chromosomes of histidine-tagged (His tag) AL1 (E) and GFP (F). TEBP-α and the wild type HSP70 chromosome were quantified as controls. Error bars for the quantitative PCR results represent standard deviation.

To determine if an artificial PCR construct could be maintained in the vegetative macronucleus, we microinjected a copy of the AL1 nanochromosome (ctg20822.0) with a 6x histidine tag fused to the carboxy-terminal of the encoded protein into the macronucleus of a clonal line in which the AL1 chromosome had been deleted (Clay *et al.* 2019). We found that the tagged chromosome was indeed present in the macronuclear genome and continued to be present after multiple asexual generations, as well as post encystment, storage at -80°, and excystment (Figure 1B, E). Other artificial constructs were similarly maintained (Table 1). To further characterize transformations, the histidine-tagged AL1 and Hsp70-GFP transformants were further investigated using the cells excysted from the encysted lines established. To quantify the copy number of the transformed construct, conventional and quantitative PCR were performed. This revealed that the constructs were maintained at a copy number within twofold of the native chromosome in wild type cells (Figure 1D, F).

**Table 1:** List of transformants generated

Transformed construct	Background strain	Details of the native chromosome that the construct derives from	Construct Details
C-terminus 6x histidine-tagged ctg20822.0.g90 (AL1) *^a^*	AL1 chromosome deletion line #5 *^b^*	Ctg20822.0 contains 1 gene: Alba-like 1	C-terminus 6x histidine tag with linker (GSGGSG) after AL1 protein
C-terminus 10x histidine-tagged ctg20822.0.g90 (AL1)	AL1 chromosome deletion line #4 *^b^*	Ctg20822.0 contains 1 gene: Alba-like 1	C-terminus 10x histidine tag with no linker after AL1 protein
N-terminus 6x histidine-tagged ctg20822.0.g90 (AL1)	AL1 chromosome deletion line #5 *^b^*	Ctg20822.0 contains 1 gene: Alba-like 1	N-terminus 6x histidine tag with linker (GSGGSG) prior to AL1 protein
C-terminus MYC-tagged ctg15169.0.g18 (AL2)	JRB310	Ctg15169.0 contains 2 genes: Alba-like 2 gene, and a predicted gene containing an EF hand motif	C-terminus MYC tag with linker (8x glycine) after AL2 protein
GFP in ctg18685.0 nanochromosome (Hsp70) *^a^*	JRB310	Ctg18685.0 contains 1 gene: Hsp70	Ciliate codon-corrected GFP replacement of the Hsp70 gene on the Hsp70 nanochromosome
Mango RNA aptamer in ctg4739.0	JRB310	Ctg4739.0 contains 1 gene containing a predicted Alba domain	Mango RNA replacement of the ctg4739.0 gene on the ctg4739.0 nanochromosome

a Transformants further characterized in this paper.

b See Clay *et al.* 2019.

While the artificial constructs are maintained after numerous generations, this does not exclude the possibility of end erosion. To test if any erosion was occurring on the introduced chromosomes, we performed telomeric PCR (Chang *et al.* 2004) and Sanger sequencing on genomic DNA harvested post encystment/excystment, representing 40 days of vegetative growth (approximately 40 generations) post-transformation. The resulting sequencing showed that the majority of GFP constructs had not had any erosion of sequence at the ends of the initial construct (see supplementary figure 1).

### Genes on transformed chromosomes are expressed similarly to native chromosomes

**Figure 2:**
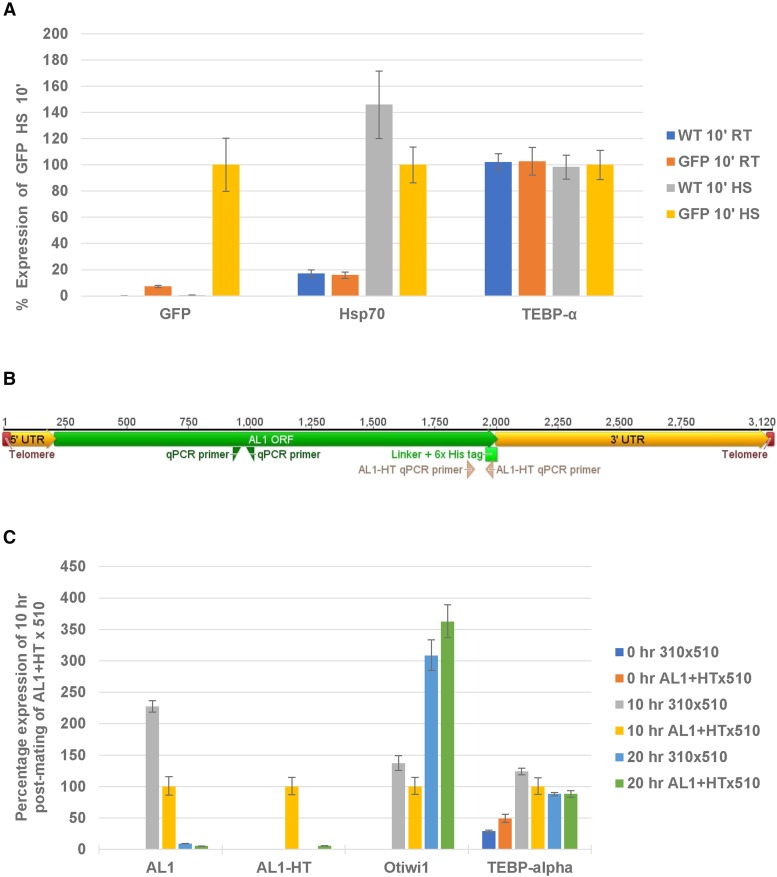
Quantitation of expression patterns of transformant and relevant native genes. A) Quantitative PCR on cDNA collected from vegetative JRB310 or the GFP transformed cells (JRB310 cells transformed with the GFP in the Hsp70 nanochromosome construct) shows the levels of GFP, HSP70, and TEBP-α expression after exposure to either heat shock for 10 min (10’ HS) or control, room temperature for 10 min (10’ RT). B) Schematic map of the AL1+His-tag construct and the location of the qPCR primer used with the AL1 primer in green and the His-tag specific qPCR primers in pink. C) Quantitative PCR on cDNA collected from JRB310 x JRB510 mated cells or from the AL1+His-tag transformant mated to JRB510 at 0, 10, and 20 hr post mixing. Error bars represent the standard deviation of the three biological replicates.

The stable transformation of the artificial chromosomes suggests that they have been functionally incorporated into the cell. To validate the artificial chromosomes’ functionality, we assayed their transcriptional activity. To investigate the tagged AL1, RNA was collected at various time points during the *Oxytricha* mating cycle. When backcrossed to the parental line JRB510, the levels of the tagged mRNA increased 100-fold during the early sexual cycle, mirroring endogenous AL1 expression (see figure 2C). To test the transcriptional activity of the Hsp70-GFP transformed constructs, the transformed line was assayed under heat shock conditions and also showed similar induction of expression after heat shock to that of the wild type Hsp70 (Figure 2A).

### Transformed chromosomes are successfully translated into expected protein products

**Figure 3:**
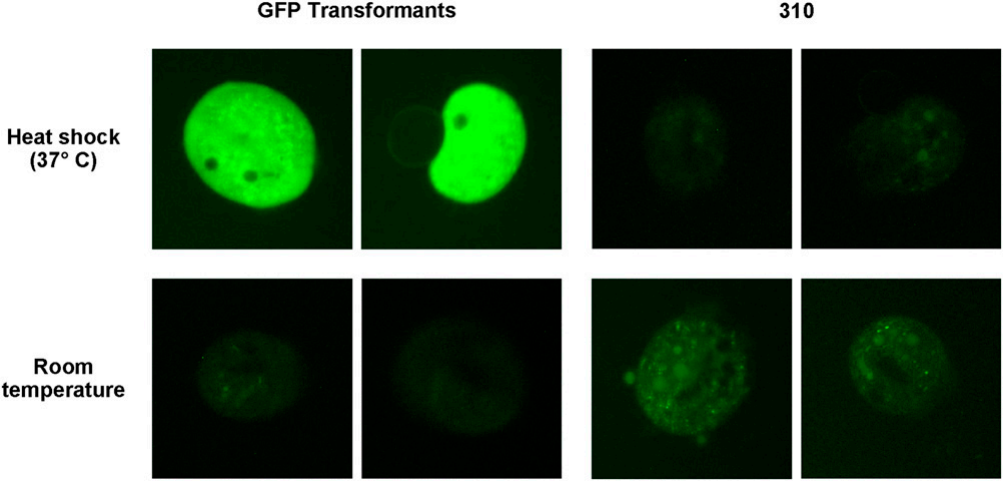
Detection of translated protein from the transformant constructs. Fluorescence microscopy of live cells immobilized under mineral oil, for the GFP transformant cell line and JRB310. Images were taken 2 hr after incubation either at room temperature or at 37° C for 20 min (heat shock), as labeled.

It has been previously reported that transformed constructs in *Euplotes* and *Stylonychia* are transcribed, and indirect evidence of translation in *Euplotes crassus* was provided through screening for transformants via neomycin selection (Bender *et al.* 1999). Using our tagged protein and GFP construct, we set out to validate if the genes present on the transformed chromosomes are successfully translated into functional proteins directly. In the case of the Hsp70-GFP transformation, heat shock resulted in detectable fluorescence within the transformed cells, while no increase in fluorescence was detected in wild type cells after heat shock (Figure 3). To verify translation of the tagged AL1 gene, a Western blot was performed on mating cells at 18 hr post mixing using an Anti-histidine tag antibody, which produced a strong signal at around 70 kilodaltons (expected size of 65 kD) in the histidine-tagged AL1 transformed line and no detectable product in the wild type cells (supplementary figure 2).

### Application of chromosome transformation to characterizing the function of AL1 protein

**Figure 4:**
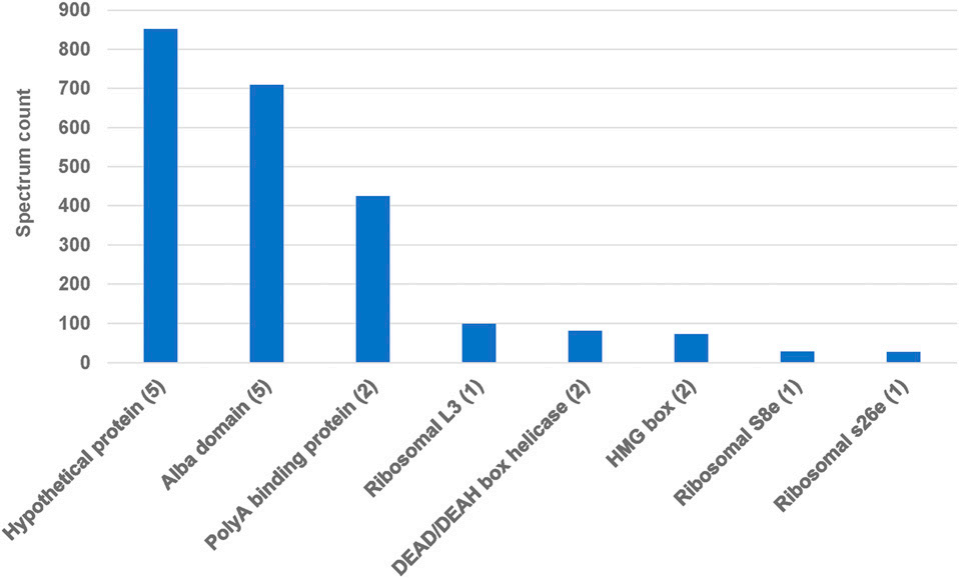
Application of the transformed AL1 tagged protein for immunoprecipitation and identification of associated proteins. Spectrum counts of protein domains from the domains identified in the top co-immunoprecipitated proteins (exclusive to the tagged sample, greater than 25 spectra counts across the three replicates, along with the presence of only one of any set of indistinguishable proteins) from AL1 pulldown by mass spectrometry. The number in parentheses represents the number of proteins in this set that contain the domain.

Having verified the translation of the AL1 histidine tagged protein, we proceeded to use the poly-histidine tag to immunoprecipitate the AL1 protein and associated proteins (Supplementary figure 3). Mass spectrometry on the immunoprecipitated elutes validated the pulldown of the targeted protein in the tagged samples, while no peptides of the AL1 protein were identified in the untagged samples. In tagged samples, a set of associated proteins was identified. Domain analysis of the co-precipitated proteins showed that the majority contained nucleic acid associated functions, with RNA binding the second most common GO term associated with the precipitates (nucleic acid binding was first). Domains identified among the abundant coprecipitates included other Alba domain proteins, PolyA binding, HMG box, and DEAD/DEAH helicase domains (Figure 4). To further investigate the property of RNA binding, RNA immunoprecipitation of the tagged protein showed enrichment for various *Oxytricha* mRNAs and RNA from the micronuclear limited 170 bp repeats, while showing a depletion of mitochondrial mRNA (supplementary figure 4).

## Discussion

### Transformed chromosomes are incorporated into the macronucleus

Here we showed that microinjected constructs can be stably maintained at high copy number in *Oxytricha* cell lines, even after encystment and excystment. To our knowledge, this is the first demonstration of the use and maintenance of artificial chromosomes in *Oxytricha*. The approach was relatively straightforward. In these transformations, the ploidy level of artificial chromosomes is similar to that of the native wild type chromosomes from which the constructs derive. The copy number maintenance may derive from an RNA-regulated mechanism for internal copy number regulation (Khurana *et al.* 2018) but selection during the PCR screen for transformants may have also introduced a bias for high copy number chromosomes. In addition to being replicated and maintained like their native counterparts, the GFP-Hsp70 synthetic chromosome shows no obvious signs of erosion at either end of the construct. This suggests that telomerase, which in *Oxytricha* is expressed during vegetative growth, is able to add the telomeric overhangs, preventing erosion during replication (Swart *et al.* 2013). Telomeric erosion depends on the length of the telomeric overhangs, suggesting a low erosion rate in *Oxytricha* due to its 16 base overhangs (Klobutcher *et al.* 1981, Huffman *et al.* 2000). In *Trypanosoma brucei*, the telomeric overhangs are reported to be less than 30 bases, and telomerase knockouts in *T. brucei* have an erosion rate of 3 to 6 base pairs per generation (Dreesen *et al.* 2005). By contrast, after approximately 40 generations, the *Oxytricha* Hsp70-GFP construct shows no change in the telomeric addition site, as also seen with the native chromosomes. We did not address whether double-stranded telomeres are required for maintenance of the transformed chromosomes, and this may reveal features of chromosome maintenance in *Oxytricha*.

### Injected synthetic chromosomes are expressed at similar levels to their native counterparts

Along with stable maintenance, the expression patterns of the transformed constructs, while not identical, are close to that of the wild type gene that the constructs replace. Transcription levels of the AL1 histidine tagged construct follow the conventional AL1 gene’s expression, with over a hundred-fold increase in expression from the 0 hr and 10 hr time points, followed by a 20-fold decline by 20 hr. Due to the high sequence identity of the AL1 tagged construct compared to the native chromosome, this recapitulation of expression suggest that transcriptional regulation is at least partly driven by DNA sequence. Expression of the GFP-Hsp70 chromosome further supports the conclusion that DNA sequence is the defining feature for transcriptional regulation in these cases, and that these features specifically reside outside of the open reading frame, as the entire Hsp70 gene has been replaced in this case, yet the GFP transcriptional activity mimics that of the native Hsp70 gene.

### Tagged proteins generated from transformed constructs provide in vivo tools for studying gene function

The tagged AL1 protein provides opportunities to investigate the protein *in vivo* that previously required custom antibodies and other resources, whereas the ability to tag a protein of interest expands the repertoire of techniques available to investigate gene function in *Oxytricha*. Due to the evolutionary distance (Parfrey *et al.* 2011) of *Oxytricha* from other model organisms, antibodies developed to target proteins in other systems are not often useful for targeting the homologous proteins in *Oxytricha*. Tagging proteins also lowers the cost and time investment for developing resources in *Oxytricha*. Furthermore, the ability to label proteins of interest may enable other tool development, such as labeling of subcellular compartments.

### Maintained macronuclear constructs and epigenetic inheritance in ciliates

One of the defining features of the dual nuclear structure of ciliates is that the somatic macronucleus is replaced during sexual development by a rearranged copy of the zygotic micronucleus. To program the conversion of the micronucleus into a new macronucleus, various RNA based pathways have been identified that transfer the informational content of the old macronucleus to the developing nucleus (reviewed in Bracht *et al.* 2013). The ability to generate synthetic constructs that differ from existing somatic chromosomes permits reproducible and scalable studies of the influence of the new or modified chromosome on genomic rearrangement.

As previously identified in *Oxytricha*, the retention of germline limited sequences along with alterations in sequence ordering in the soma are epigenetically inherited in the next generation (Nowacki *et al.* 2008, Fang *et al.* 2012). Transformation will permit further investigation of the parameters of somatic epigenetic transfer, using artificial somatic chromosomes to test their ability to program novel rearrangements, similar to the DNA injections in Nowacki *et al.* (2008), but by mating transformed lines to wild type cells, obviating the need for direct manipulation of cells during early development. Transformation also permits the ability to alter defined strains, whereas template or piRNA injection (Nowacki *et al.* 2008, Fang *et al.* 2012) produces the initial alteration in the F1 progeny of the injected mating cells (typically a cross between strains JRB310 x JRB510).

## Conclusions

### Transformation in Oxytricha

The development of somatic transformations in *Oxytricha* opens many opportunities to study the general biology of *Oxytricha* as well as the process of genome rearrangement. The successful incorporation of artificial chromosomes into the macronuclear genome, with no degradation, as demonstrated here, contributes to the expanding toolkit for this model system.
